# Defect symmetry influence on electronic transport of zigzag nanoribbons

**DOI:** 10.1186/1556-276X-6-254

**Published:** 2011-03-24

**Authors:** Hui Zeng, Jean-Pierre Leburton, Yang Xu, Jianwei Wei

**Affiliations:** 1 College of Physical Science and Technology, Yangtze University, Jingzhou, Hubei 434023, China; 2 Beckman Institute for Advanced Science and Technology, University of Illinois at Urbana-Champaign, Urbana, IL 61801, USA; 3 Department of Electrical and Computer Engineering, University of Illinois at Urbana-Champaign, Urbana, IL 61801, USA; 4 Department of Physics, University of Illinois at Urbana-Champaign, Urbana, IL 61801, USA; 5 Department of Information Science and Electronic Engineering, Zhejiang University, Hangzhou, Zhejiang 310027, China; 6 College of Mathematics and Physics, Chongqing University of Technology, Chongqing 400054, China

## Abstract

The electronic transport of zigzag-edged graphene nanoribbon (ZGNR) with local Stone-Wales (SW) defects is systematically investigated by first principles calculations. While both symmetric and asymmetric SW defects give rise to complete electron backscattering region, the well-defined parity of the wave functions in symmetric SW defects configuration is preserved. Its signs are changed for the highest-occupied electronic states, leading to the absence of the first conducting plateau. The wave function of asymmetric SW configuration is very similar to that of the pristine GNR, except for the defective regions. Unexpectedly, calculations predict that the asymmetric SW defects are more favorable to electronic transport than the symmetric defects configuration. These distinct transport behaviors are caused by the different couplings between the conducting subbands influenced by wave function alterations around the charge neutrality point.

## Introduction

As a truly two-dimensional nanostructure, graphene has attracted considerable interest, mainly because of its peculiar electronic and transport properties described by a massless Dirac equation [1,2]. As such, it is regarded as one of the most promising materials since its discovery [3-5] because charge carriers exhibit giant intrinsic mobility and long mean-free path at room temperature [6,7], suggesting broad range of applications in nanoelectronics [8-11]. Several experimental [4,8,12,13] and theoretical [2,14,15] studies are presently devoted to the electronic, transport, and optical properties [16] of graphene. By opening an energy gap between valence and conduction bands, narrow graphene nanoribbons (GNR) are predicted to have a major impact on transport properties [17,18]. Most importantly, GNR-based nano-devices are expected to behave as molecular devices with electronic properties similar to those of carbon nanotubes (CNTs) [19,20], as for instance, Biel et al. [21] reported a route to overcome current limitations of graphene-based devices through the fabrication of chemically doped GNR with boron impurities.

The investigation of transport properties of GNRs by various experimental methods such as vacancies generation [22], topological defects [23], adsorption [24], doping [25], chemical functionalization [26-28], and molecular junctions [29] have been reported. Meanwhile, defective GNR with chemically reconstructed edge profiles also have been experimentally evidenced [30] and have recently received much attention [31,32]. In particular, Stone-Wales (SW) defects, as one type of topological defects, are created by 90° rotation of any C-C bond in the hexagonal network [33], as shown by Hashimoto et al. [34]. More recently, Meyer et al. [35] have investigated the formation and annealing of SW defects in graphene membranes and found that the existence of SW defects is energetically more favorable than in CNTs or fullerenes. Therefore, the influences of SW defects on electronic transport of GNRs is crucial for the understanding of the physical properties of this novel material and for its potential applications in nanoelectronics.

In this brief communication, we investigate the influence of SW defects on the electronic transport of zigzag-edged graphene nanoribbons (ZGNRs). It is found that the electronic structures and transport properties of ZGNRs with SW defects can very distinctively depend on the symmetry of SW defects. The transformation energies obtained for symmetric SW defects and asymmetric SW defects are 5.95 and 3.34eV, respectively, and both kinds of defects give rise to quasi-bound impurity states. Our transport calculations predict different conductance behavior between symmetric and asymmetric SW defects; asymmetric SW defects are more favorable for electronic transport, while the conductance is substantially decreased in the symmetric defects configuration. These distinct transport behaviors result from the different coupling between the conducting subbands influenced by the wave function symmetry around the charge neutrality point (CNP).

## Model and methods

The optimization calculations are done by using the density functional theory utilized in the framework of SIESTA code [36,37]. We adopt the standard norm-conserving Toullier-Martins [38] pseudopotentials orbital to calculate the ion-electron interaction. The numerical double-*ζ *polarized is used for basis set and the plane cutoff energy is chosen as 200 Ry. The generalized gradient approximation [39] proposed by Perdew and Burke and Ernzerhof was employed to calculate exchange correction term. All nanostructure geometries were converged until no forces acting on all atoms exceeded 0.01eV/Å.

**Figure 1:**
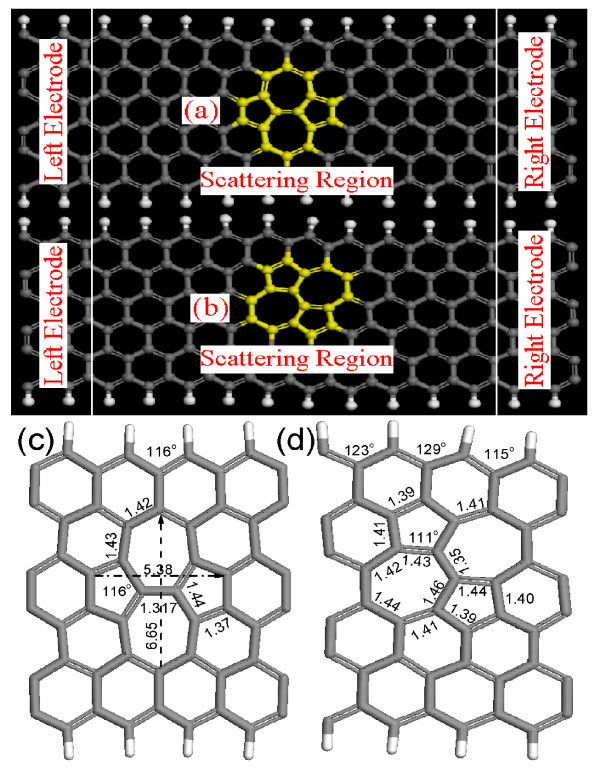
**(Color online) Schematics of the molecular device considered in the calculation of ZGNR**. The whole device is composed of scattering region and two electrodes containing the corresponding pristine ZGNRs. The SW defects are highlighted by yellow atoms **(a) **ZGNR with symmetric SW defects, some C-C bond lengths and angles are shown by **(c)**; **(b) **ZGNR with asymmetric SW defects, while some C-C bond lengths and angles are shown by **(d)**.

The electronic transport properties of the nanoribbon device have been performed by using non-equilibrium Green's function (NEGF) methodology [40,41]. In order to self-consistently calculate the electrical properties of nanodevices, we construct the two-probe device geometry where the central region contains the SW defects and both leads consist each of the two supercell pristine ZGNR, as shown in Figure 1. The equilibrium conductance *G *is obtained from the Landauer formula such that *G *= *G*_0_*T *(*E*), where *G*_0 _is the quantum conductance with relationship . The transmission coefficient *T *as a function of the electron energy *E *is given by(1)

where *Σ^l ^*(*Σ^r^*) represents the self-energies of the left (right) electrode, *G*^R ^(*G*^A^) is retard (advanced) Green's function. It is calculated from the relation:(2)

where *H*_S _is the Hamiltonian of the system. More details about the NEGF formalism can be found in Ref. [42].

In this study, we consider symmetric and asymmetric SW defects contained in 6-ZGNRs, where 6 denotes the number of zigzag chains (dimers) across the ribbon width [18]. Taking into account screening effects between electrodes and central molecules, we use 10-unit cell's length as scattering regions, and 2 units as electrodes to perform transport calculation. The electron temperature in the calculation is set to be 300 K.

## Results and discussions

In Figure 1, we show the geometry of defective ZGNR after relaxation. After introducing symmetric SW defects, the GNR shrinks along the width axis, by 0.526 Å, and correspondingly, the nearest four H atoms move toward the central region by 0.21 Å. As a result, the bond angles of the edge near the SW defects are reduced from 120 to 116°, as shown in Figure 1c, d. In contrast to the shrinking along the width axis, the SW defects stretch from 4.88 to 5.38 Å along the length axis direction. No distinct change for the H-C bond length at the edge is observed. Thus, the effect of symmetric SW defects on the geometry modification is limited to the defective area, with mirror reflection around their axis. However, the presence of asymmetric SW defects to the geometry modification is far more complex. They twist the whole structure by shifting the left side upward, while the right side is downward shifted. Hence, the mirror symmetry is broken because of the asymmetric SW. The transformation energies for symmetric and asymmetric SW defects are 5.95 and 3.34eV, respectively. These results imply that the asymmetric SW defects are energetically more favorable than the symmetric SW defects.

**Figure 2:**
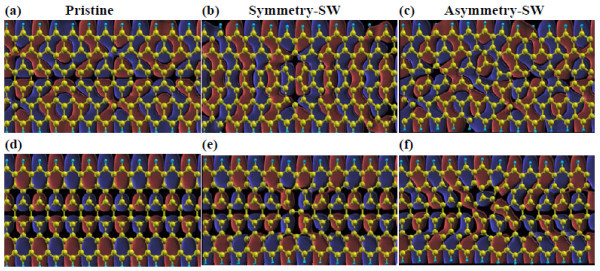
**(Color online) Wave functions at the Gamma point of defective ZGNRs**. Wave functions at the Gamma point associated with the LUES above the CNP (top Panel) and the HOES below the CNP (bottom Panel) for ZGNR with no defects **(a, d)**, symmetric SW defects **(b, e)** asymmetric SW defects **(c, f)**. Dark gray (blue online) and light gray (red online) colors correspond to the opposite signs of the wave function.

Wave functions of electronic states at the Gamma point of the highest-occupied electronic states (HOES) and the lowest-unoccupied electronic states (LUES) are depicted in Figure 2. As expected, the wave functions of the pristine even-index ZGNR at the Gamma-point associated to the HOES and LUES exhibit the well-defined parity with respect to the mirror plane, and their eigenstates in the case of symmetric SW defects, the HOES and LUES, keep the same parity because of the potential induced by the symmetric defects [25,43]. Note that, although the wave functions of both the pristine and symmetric SW defects have well-defined parity, the sign of their wave functions, especially for the electronic states below the CNP, are precisely opposite. For the asymmetric SW defects, the well-defined parity of the wave functions is not preserved. Moreover, the wave function symmetry in this configuration is broken leading to substantial electron backscattering below and above the CNP.

**Figure 3:**
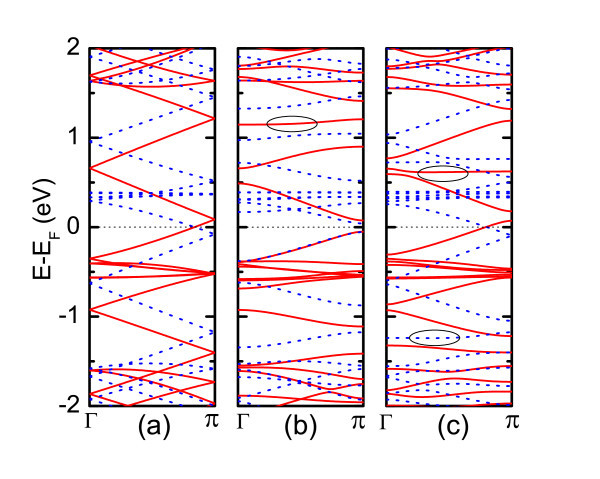
**(Color online) Electronic band structures of defective ZGNRs**. **(a) **for the pristine, **(b) **for the symmetric SW defects and **(c) **for the asymmetric SW defects. The solid red (dotted blue) line denotes the *α*-spin (*β*-spin) bands. The dashed black line indicates the CNP, and solid circles indicate defect states.

The central issue of this study is to investigate the influence of SW defects in the ZGNRs on their electronic and transport behavior. ZGNRs are known to present very peculiar electronic structure, that is, strong edge effects at low energies originated from the wave functions localized along the GNR edges [44]. Spin-unpolarized calculations reveal that all ZGNRs are metallic with the presence of sharply localized edge states at the CNP [25,43,44], while *ab initio *calculation with spin effect taken into consideration found that a small band gap opens up [18]. The electronic band structures of defective nanoribbons and the corresponding pristine GNRs are shown for comparison. In the case of pristine GNR, zone-folded effects give rise to nondegenerated bands for *α*- and *β*-spin states, and the corresponding spin bands shift upward and downward with respect to the CNP, respectively. It also leads to gapless electronic structure as well as 3*G*_0 _conductance in the vicinity of CNP (see Figure 3). Meanwhile, zone-folded effects create more subbands near the CNP, namely, four *α*-spin subbands around 0.4eV and four *β*-spin subbands around 0.4eV. The presence of symmetric SW defects substantially split the electronic bands, especially for the *β*-spin bands above the CNP, resulting from the bands anticrossing at *Γ *or *π *point. More importantly, the symmetric defects open a band gap of about 0.12eV for *α*-spin bands and 0.09eV for *β*-spin bands, which is attributed to the mismatch coupling between its LUES and HOES wave functions due to the presence of defects. It is interesting to note that a defect state deriving from the *α*-spin subband is located at about 1.15eV above the CNP producing a localized state, where complete backscattering is obtained (see red dashed line in Figure 3). Thus, these changes in the band structures arising from introducing symmetric SW defects are unfavorable to electronic transport. In contrast to the extensive split produced by the symmetric SW defects, the electronic structure modification due to the asymmetric SW defects is slight. Except for some bands splitting that could be unfavorable to electron transport, the band structure away from the CNP does not experience much change. Similar to the emergence of defect states induced by the symmetric SW configuration, two defect states are observed in the asymmetric SW configurations; one defect state arising from the *α*-spin subband locates at about 0.62eV above the CNP, and the other one from the *β*-spin is -1.20eV below the CNP. Both defects give rise to localized states that lead to conductance gaps (see, dotted line in Figure 3). Overall, the band structure results reveal that the SW defect states near the CNP lead to complete electron backscattering region, where the location depends on the spatial symmetry of the defects.

**Figure 4:**
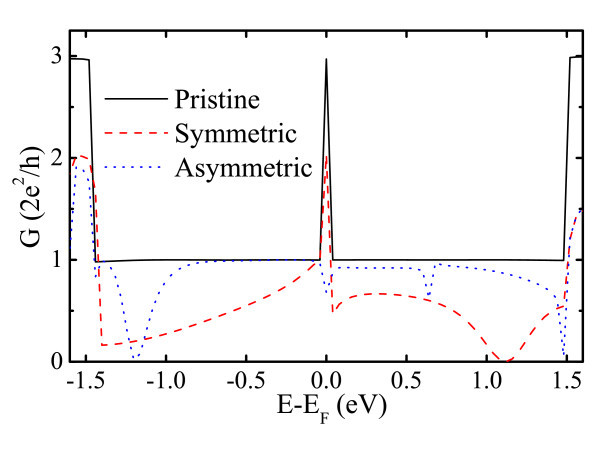
**(Color online) Conductance of defective ZGNRs as a function of electron energy**. The black solid line, red dashed line, and blue dotted line denote the results of pristine, symmetric SW, and asymmetric SW defects.

The electronic transport results are displayed in Figure 3. The states induced by H atoms at the edge produce a conductance peak in the vicinity of CNP in the pristine ZGNR. In this study, our results show a good agreement with previous studies [43-45]. The first conductance plateau corresponding to the occupied and unoccupied states is *G*_0_. In the case of symmetric SW defects in the ZGNR, the conductance in the vicinity of CNP is decreased as a result of the four H atoms shrinking. The conductance with symmetric defects remarkably decreases below the CNP, manifesting monotonous reduction of conductance with increasing electron energy. We attribute this effect to the antisymmetry (opposite sign at every position) of wave functions, with respect to the pristine GNR in the wave functions (see Figure 4e) that block the electronic transport. On the other hand, the orientation of about 50% of all wave functions corresponding to LUES is reversed, which gives rise to a conducting plateau (about 0.5*G*_0_) that ranged from 0.04 to 0.8eV above the CNP. More importantly, strong electron backscattering induced by the coupling between all states are expanded to lead to full suppression of the conduction channel at particular resonance energies. Accordingly, a smooth conductance valley around 1.12eV corresponding to complete electron backscattering is observed. Concerning the transport properties of asymmetric SW configuration, we find that the absence of conductance peak at the CNP is due to the breaking of edge states. In addition, localized states in the vicinity of CNP lead to reduced conductance. The main feature of the first conducting plateau below the CNP is preserved except for the smooth conductance valley located at about -1.2eV. This illustrates the obvious different transport behaviors between the symmetric and asymmetric SW defects. We indeed found that such different transport behaviors result from different coupled electronic states supported by the wave function results. The HOES and LUES wave functions of asymmetric SW defect configuration are very similar to that of the pristine GNR except for the defective area. Therefore, the first conducting plateau near the CNP is preserved for the asymmetric configuration. Naturally, the asymmetric SW defects are responsible for the two conductance valleys, namely, a smooth valley at -1.2eV and a sharp valley at 1.48eV. The large reduction of conductance at these areas induced by the asymmetric SW defects corresponds to complete electron backscattering region. which is different from the situation in CNTs, where SW defects induce suppression of only half of the conductance channels [46]. However, the impact of the two conductance valleys on the ZGNRs is limited because they are far away from the CNP. The transport properties of asymmetric SW configuration are predicted to be comparable with that of the pristine GNR in spite of non-preservation of the geometry and wave function symmetry for the former. We note that similar results have been obtained under spin-dependent calculation by Ren et al. [47] very recently. Overall, the electronic transport calculations predict that it is more likely to be observed for asymmetric SW defects in the ZGNR, since these defects are more favorable for electronic transport in contrast to the substantially transport degradation in the symmetric defects configuration.

## Conclusion

In summary, we investigate the influence of local structural defects on the electronic transport of ZGNR using first principles calculations. The transformation energies reveal that the asymmetric SW defects is energetically more favorable than the symmetric SW defects. Both defects give rise to complete electron backscattering region that depends on the spatial symmetry of the defects. Our transport calculations predict that the asymmetric SW defects are more favorable for electronic transport in contrast to the substantially decreased in the symmetric defects configuration. We attribute these distinct transport behaviors to the different coupling between the conducting subbands influenced by the wave function modification around the CNP.

## Abbreviations

CNP: charge neutrality point; GNR: graphene nanoribbons; HOES: highest-occupied electronic states; LUES: lowest-unoccupied electronic states; SW: Stone-Wales; ZGNR: zigzag-edged graphene nanoribbon.

## Competing interests

The authors declare that they have no competing interests.

## Authors' contributions

HZ carried out molecular dynamic studies, participated in the sequence alignment and drafted the manuscript. JL participated in the design of the study and the sequence alignment. YX participated in the sequence alignment. JW took part in the simulation and participated in the sequence alignment. All the authors discussed the results.
